# Complete mitochondrial genome of the spotted-wing drosophila, *Drosophila suzukii* (Diptera: Drosophilidae)

**DOI:** 10.1080/23802359.2016.1155422

**Published:** 2016-03-28

**Authors:** Jong Seok Kim, Min Jee Kim, Deuk-Soo Choi, Iksoo Kim

**Affiliations:** a College of Agriculture & Life Sciences, Chonnam National University, Gwangju, Republic of Korea;; b Department of Plant Quarantine, Animal and Plant Quarantine Agency, Anyang, Republic of Korea

**Keywords:** *Drosophila suzukii*, invasive pest, mitochondrial genome

## Abstract

The spotted-wing drosophila, *Drosophila suzukii* (Diptera: Drosophilidae) is an Asian species introduced into North America and Europe. It damages a wide variety of thin-skinned fruits. We sequenced the complete mitochondrial genome (mitogenome) to better understand the mitogenomic characteristics of this species. The 16 230-bp complete mitogenome of the species consists of a typical set of genes, including 13 protein-coding genes (PCGs), two rRNA genes and 22 tRNA genes, and one major non-coding A + T-rich region, with an arrangement typical of insects. Twelve PCGs began with the typical ATN codon, whereas the COI began with TCG, which has been designated as the start codon for other *Drosophila* species. The 1525-bp A + T-rich region is the second longest in *Drosophila* species for which the whole mitogenome has been sequenced, after *D. melanogaster*. Phylogenetic analysis using the 13 PCGs of the *Drosophila* species indicated that *D. suzukii* is placed, with a strong support, in the basal lineage of the previously defined Melanogaster group.

*Drosophila suzukii* (Diptera: Drosophilidae) is an economically damaging pest. The females have serrated ovipositor, which enables them to pierce most thin-skinned fruits (Walsh et al. 2011). *D. suzukii* is an Asian species that has been recorded in China, India, Japan, South Korea and Thailand (Hauser 2011), but has invaded the United States, British Columbia, Italy, France and Spain (Toda 1991; Hauser et al. 2009; Lee et al. 2011).

In this study, we sequenced the complete mitochondrial genome (mitogenome) of *D. suzukii* to better understand the mitogenomic characteristics of this species and its phylogenetic relationships within *Drosophila*. One adult was captured from an arboretum located in Gwangju City, South Korea (35°10′21.6″ N, 126°53′57.6″ E). A voucher specimen was deposited in Chonnam National University, Gwangju, Korea.

By using the total DNA as template, two long overlapping fragments (COI–CytB and CytB–COI) were amplified, and subsequently, 26 short overlapping fragments were amplified using the two long fragments as templates. Primers for the long and short fragments were designed in this study by using available *Drosophila* mitogenome sequences (Andrianov et al. 2010; De Ré et al. 2014; Llopart et al. 2014).

The *D. suzukii* mitogenome is 16 230 bp in length and includes the typical sets of genes (two rRNAs, 22 tRNAs and 13 protein-coding genes [PCGs]) and a major non-coding A + T-rich region (GenBank accession number: KU588141). The mitogenome size is well within the range found in *Drosophila*, from 15 450 bp (*D. incompta*; De Ré et al. 2014) to 19 317 bp (*D. melanogaster*; Unpublished, GenBank accession number: KJ947872). The 1525-bp A + T-rich region is the second longest among sequenced *Drosophila*, after the 4608-bp A + T-rich region of *D. melanogaster*. However, despite the length, the *D. suzukii* A + T-rich region does not have long tandem repeats. Instead, it has interspersed TA repeat, poly-T stretch and poly-A stretch. The gene arrangement of *D. suzukii* is identical to that of the ancestral insect order found in majority of insects (Boore 1999).

The AT content among genes and regions varies profoundly in the *D. suzukii* mitogenome, including the A + T-rich region (92.5%), *lrRNA* (82.7%), *srRNA* (78.9%), *trns* (75.9%) and PCGs (76.23%). Twelve *D. suzukii* PCGs began with the typical ATN codon (six with ATG, four with ATT, one with ATC and one with ATA), whereas COI began with the atypical sequence TCG (Serine), as has been proposed in other species such as *D. littoralis*, *D. pseudoobscura*, *D. santomea*, *D. yakuba* and *D. melanogaster* (Clary & Wolstenholme 1985; Torres et al. 2009; Andrianov et al. 2010; Llopart et al. 2014; Unpublished, GenBank accession number: KJ947872).

We performed a phylogenetic analysis by using the 13 PCGs and the available mitogenome sequences of 11 *Drosophila* downloaded from GenBank and two species of Tephritidae as outgroups. Bayesian inference method was performed using the GTR + GAMMA + I model in CIPRES Portal v. 3.1 (Miller et al. 2010). The results showed that *D. suzukii* is placed as the basal lineage of the previously defined Melanogaster group (Da Lage et al. 2007), with a strong support (Bayesian posterior probabilities = 1.0). The Melanogaster group included the following species: *D. sechellia*, *D. simulans*, *D. mauritiana*, *D. melanogaster*, *D. santomea* and *D. yakuba* (Figure 1).

**Figure 1.**
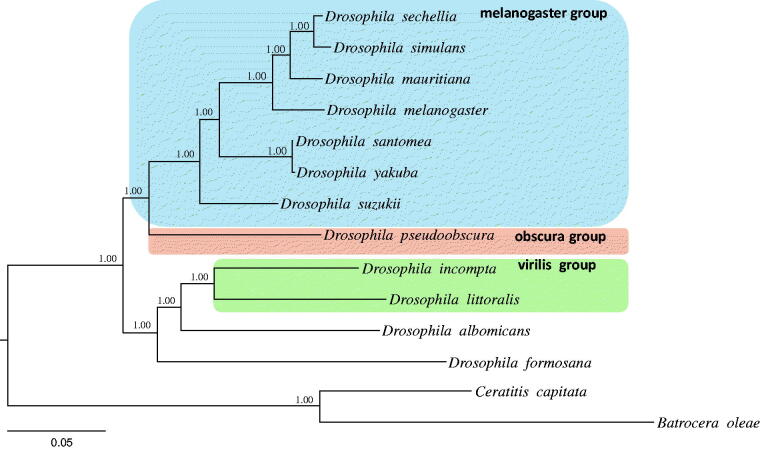
Phylogeny of *Drosophila* constructed using the Bayesian inference method by analyzing 13 protein-coding genes (PCGs). Values at each node specify Bayesian posterior probabilities in percent. The scale bar indicates number of substitutions per site. Two species of Tephritidae were included as outgroups. GenBank accession numbers are as follows: *D. suzukii*, KU588141; *D. albomicans*, KT119344; *D. incompta*, KM275233; *D. littoralis*, FJ447340; *D. pseudoobscura*, FJ899745; *D. mauritiana*, AF200830; *D. santomea*, KF824856; *D. sechellia*, AF200832; *D. simulans*, AF200833; *D. yakuba*, NC_001322; *D. melanogaster*, KJ947872; *D. formosana*, KR265324; *Ceratitis capitate*, NC_000857.1 and *Bactrocera oleae*, NC_005333.1.
